# O013. Neuro-imaging and history of cases of refractory chronic cluster headache in young patients: a hint for reflections

**DOI:** 10.1186/1129-2377-16-S1-A95

**Published:** 2015-09-28

**Authors:** Maria Nicolodi, Anna Torrini, Vanessa Sandoval, Manfredo Fanfani, Ivano Taddei

**Affiliations:** Foundation Prevention and Therapy Primary Pain and Headache, Florence, Italy

## Background

Hypothalamus has been suggested to be the crucial area of the brain for the stemming off of cluster headache (CH) attacks[1–3].

## Aim

To verify activation of brain areas during attacks in chronic, refractory young CH sufferers.

## Materials and methods

The observation included 6 patients (6 males; aged 18-21 years). The observation started in February 2011. Inclusion criteria were: diagnosis of chronic CH according to the ICHD-II, and patient refractory to any prophylactic and acute abortive treatment. In all the patients vegetative signs, characteristic of CH attack, were not observed, whilst VAS and behaviour measures showed excruciating pain. Exclusion criteria were: psychiatric illness (DMS IV parameters), epilepsy, CNS pathology evidenced with MRI. All the volunteers overused both sumatriptan and fentanyl, when they presented to our structure. Fentanyl and sumatripan gave very moderate benefits (mean 1.5, 05%±05.5 SD on 0-10 VAS). The abuse lasted over 1 year (mean 3.6 years±1.2 SD). Nevertheless, it was impossible to obtain a real dis-habituation in these young sufferers. All patients, as well as one or both their parents, reported headache onset when the patient was 6 to 10 years old. Diagnosis of migraine without aura was made in accordance with the IHS criteria. It lasted for a period of 3-10 months (mean 4.1±4.7 SD). Furthermore, migraine switched to CH and became chronic in a very short period (mean 3.3±1.4 month SD). During the migraine period the 6 young patients underwent a SPECT observation during attack. In all the cases, thalamic hypoperfusion was evidenced at the contro-lateral side where CH manifested, another symptom was a moderate ipsilateral hypoperfusion at the frontal level. We performed (18) FDG/PET-CT, where CT implemented attenuation-corrected images and better defined anatomo-structural localization.

## Results

The PET-CT examination evidenced hypometabolism at the level of the thalamus on the same side of cluster pain. The datum, together with the previous SPECT outcomes, supports the suggestion that a thalamic genesis is a possible origin of refractory CH.

## Conclusions

It seems that the use of imigran sumatriptan and fentanyl may have induced some variations in receptoral binding affinity which is not expected to dramatically change anatomic area of activation during the stemming off of CH attacks. This may suggest verifying the focus area in refractory CH, leading us to consider alternative therapy.

Written informed consent to publication was obtained from the patient(s).
